# Apolipoprotein A1 Enhances Endothelial Cell Survival in an *In Vitro* Model of ALS

**DOI:** 10.1523/ENEURO.0140-22.2022

**Published:** 2022-07-27

**Authors:** Svitlana Garbuzova-Davis, Alison E. Willing, Cesario V. Borlongan

**Affiliations:** 1 Center of Excellence for Aging & Brain Repair, University of South Florida, Morsani College of Medicine, Tampa, FL 33613; 2 Department of Neurosurgery and Brain Repair, University of South Florida, Morsani College of Medicine, Tampa, FL 33613; 3 Department of Molecular Pharmacology and Physiology, University of South Florida, Morsani College of Medicine, Tampa, FL 33613; 4 Department of Pathology and Cell Biology, University of South Florida, Morsani College of Medicine, Tampa, FL 33613

**Keywords:** ALS SOD1 mice, apolipoprotein A1, endothelial cell, *in vitro*, LIVE/DEAD cell viability assay

## Abstract

Altered lipoprotein metabolism is considered a pathogenic component of amyotrophic lateral sclerosis (ALS). Apolipoprotein A1 (ApoA1), a major high-density lipoprotein (HDL) protein, is associated with prevention of vascular damage. However, ApoA1’s effects on damaged endothelium in ALS are unknown. This study aimed to determine therapeutic potential of ApoA1 for endothelial cell (EC) repair under a pathologic condition reminiscent of ALS. We performed *in vitro* studies using mouse brain ECs (mBECs) exposed to plasma from symptomatic G93A SOD1 mice. Dosage effects of ApoA1, including inhibition of the phosphoinoside 3-kinase (PI3K)/Akt signaling pathway and integration of ApoA1 into mBECs were examined. Also, human bone marrow-derived endothelial progenitor cells (hBM-EPCs) and mBECs were co-cultured without cell contact to establish therapeutic mechanism of hBM-EPC transplantation. Results showed that ApoA1 significantly reduced mBEC death via the PI3K/Akt downstream signaling pathway. Also, ApoA1 was incorporated into mBECs as confirmed by blocked ApoA1 cellular integration. Co-culture system provided evidence that ApoA1 was secreted by hBM-EPCs and incorporated into injured mBECs. Thus, our study findings provide important evidence for ApoA1 as a potential novel therapeutic for endothelium protection in ALS. This *in vitro* study lays the groundwork for further *in vivo* research to fully determine therapeutic effects of ApoA1 in ALS.

## Significance Statement

Altered lipoprotein metabolism is one pathogenic component in amyotrophic lateral sclerosis (ALS). Apolipoprotein A1 (ApoA1) may prevent vascular damage. However, ApoA1’s effects on injured CNS endothelial cells (ECs) in ALS are unclear. This study was designed to determine the potential impact of ApoA1 for EC repair under an ALS-like pathologic condition. To explore therapeutic mechanism of ApoA1, mouse brain ECs (mBECs) were cultured with plasma from symptomatic mice modeling ALS. Also, human bone marrow-derived endothelial progenitor cells (hBM-EPCs) and mBECs were co-cultured. ApoA1 significantly reduced mBEC damage by adsorption into mBECs. Coculture provided evidence that ApoA1 was secreted by hBM-EPCs and incorporated into injured mBECs. Study findings identify ApoA1 as a potential novel therapeutic for endothelium repair in ALS.

## Introduction

Dyslipidemia in amyotrophic lateral sclerosis (ALS), a fatal neurodegenerative disorder, is considered an essential component of the pathologic disease process (Joardar et al., 2017; González De Aguilar, 2019; Chełstowska et al., 2021). Alterations in the lipid and apolipoprotein metabolisms were noted in association with ALS risk even decades before disease diagnosis (Mariosa et al., 2017). The authors showed that ALS patients had increased serum levels of low-density lipoprotein (LDL), high-density lipoprotein (HDL), apolipoprotein B (ApoB), and apolipoprotein A1 (ApoA1) before ALS diagnosis. Also, gradually declining LDL/HDL and ApoB/ApoA1 ratios during the years before ALS diagnosis were noted without major increases in either LDL or ApoB levels. Although association of higher lipid blood levels with longer patient survival after ALS diagnosis was reported (Mandrioli et al., 2017; Ahmed et al., 2018; Ingre et al., 2020), some studies were contradictory regarding hyperlipidemia in ALS patients, showing no link between lipids and ALS survival (Chiò et al., 2009; Paganoni et al., 2011; Rafiq et al., 2015). However, it is possible that lipid disturbance in ALS patients reflects requirements of particular lipid(s) because of cellular energy demands for survival. In support of this possibility, hyperlipidemia, especially high plasma levels of total cholesterol or LDL/HDL ratio, was associated with an increase in ALS patient survival of >12 months (Dupuis et al., 2008). In addition, a recently reported transcriptomic meta-analysis of the spinal cords from G93A SOD1 mice confirmed lipid metabolism dysregulation as an early pathologic disease mechanism (Fernández-Beltrán et al., 2021), supporting previous observations in a mouse model of ALS and ALS patients (Chaves-Filho et al., 2019; González De Aguilar, 2019).

Lipoprotein metabolism in plasma is regulated and controlled by apolipoprotein (Apo) constituents such as ApoE, ApoB, ApoA1, ApoAII, ApoAIV, ApoCI, ApoCII, and ApoVIII through their involvement in the transport and distribution of lipids among various cells and tissues under healthy and pathologic conditions (Mahley et al., 1984; Koschinsky and Marcovina, 2004). Particularly, ApoB, a component of LDL, is involved in dyslipidemia and atherogenesis (Benn, 2009; Morita, 2016) while ApoA1, an HDL component, provides antioxidative, anti-inflammatory, and antithrombotic actions preventing vascular damage (Walldius, 2012). Moreover, the ApoB/ApoA1 ratio demonstrated a stronger association with vascular risk than either lipids, lipoprotein levels, or lipid ratios (for review, see Walldius and Jungner, 2006; Schmidt and Bergstrom, 2014). Also, it has been shown that high ApoB levels and high ApoB/ApoA1 ratios were strongly related to increased coronary risk, whereas high ApoA1 levels indicated decreased risk of coronary events (Walldius et al., 2004). Since ApoB/ApoA1 ratio primarily reflects the balance between proatherogenic and anti-atherogenic lipoproteins (Walldius, 2012), imbalance between these apolipoproteins may be an etiologic mechanism for ALS (Mariosa et al., 2017).

ApoA1, as the major protein component of HDL, exerts multiple functions in treatment of several diseases such as atherosclerosis, thrombosis, diabetes, and other cardiovascular related detriments (Y. Wang et al., 1998; Mineo et al., 2006; Tabet and Rye, 2009; Smith, 2010; W. Wang et al., 2010; Cochran et al., 2021). Beneficial effects of ApoA1 were also demonstrated on inhibition of endothelial cell (EC) and arterial inflammation *in vitro* and *in vivo* (Shah et al., 1998, 2001; Yan et al., 2006; Jiao and Wu, 2008; Y. Li et al., 2008; Puranik et al., 2008; Svensson et al., 2017). ApoA1 has been found to reduce inflammation in ECs by increasing annexin A1 expression and inhibiting the activation phospholipase A_2_ (Pan et al., 2016). Downstream signaling pathways were investigated for mediation of ApoA1-induced cell responses and showed that ApoA1 upregulated angiopoietin 4 (ANGPTL4) expression in human aortic ECs via the phosphoinoside 3-kinase (PI3K)/Akt/FOX01 signaling pathway (Theofilatos et al., 2018). It has also been reported that ApoA1 induced proliferation and angiogenic capacity of human ECs *in vitro* through activation of the ecto-F1-ATPase receptor via downstream PI3Kβ pathway (González-Pecchi et al., 2015; Castaing-Berthou et al., 2017). In another study, blockage of the PI3K/Akt-dependent signaling pathway with wortmannin prevented microvesicle-induced angiogenesis, likely because of inhibiting transfer of proteins to ECs (Deregibus et al., 2007).

Despite the evidence that ApoA1 has an advantageous role in stabilization of EC maintenance, the effects of this protein on the ALS-damaged CNS endothelium in both patients (Garbuzova-Davis et al., 2012) and an animal model of disease (Garbuzova-Davis et al., 2007a,b) are unknown.

The aim of this study was to determine the ApoA1 effects on ECs under a pathologic environmental condition reminiscent of ALS *in vitro*. A specific focus was examining the inhibition of the PI3K/Akt signaling pathway and blockage of ApoA1 integration in damaged ECs in development of ApoA1 as a potential therapeutic agent for endothelium repair in ALS.

## Materials and Methods

### Ethics

Blood was collected from G93A SOD1 mutant male mice and control noncarrier mutant SOD1 gene male mice used in our previously published studies (Garbuzova-Davis et al., 2017, 2019b). All described procedures for animals used in these studies were approved by our university’s Institutional Animal Care and Use Committee and conducted in compliance with the *Guide for the Care and Use of Laboratory Animals*. All applicable international, national, and/or institutional guidelines for the care and use of animals were followed.

### Cell preparation and culture procedure

#### Mouse brain ECs (mBECs)

Cryopreserved mBECs from line bEnd.3 [BEND3] (ATCC CRL-2299, RRID:CVCL_0170; mBECs; ATCC) were used as previously described (Garbuzova-Davis et al., 2020). Briefly, the cells were thawed and centrifuged (130 × *g*/7 min) with PBS 1×, pH 7.2 (catalog #SH30256.01, HyClone Laboratories) at room temperature (RT), the supernatant discarded, and the process repeated. After the final wash, cell viability was assessed before culturing as described above. The mBECs were cultured in ATCC-formulated DMEM (catalog #30-2002) containing 10% fetal bovine serum (FBS; Corning, catalog #35-010-CV, lot #35010161).

The mBECs were cultured in a 24-well plate (2 × 10^4^ cells/500 μl media/well) for 48 h, reaching 80–85% cell confluence. Media was then changed by adding 3% ALS plasma obtained from early symptomatic G93A SOD1 mutant mice for an additional 48 h incubation. The mBECs were then incubated in media supplemented with 3% ALS mouse plasma and ApoA1 from human plasma with low endotoxin levels (Athens Research & Technology, catalog #16-16-120101-LEL) at a concentration of 10 μg/ml, 50 μg/ml, or 100 μg/ml in duplicate. In a separate culture set, mBECs were incubated in media supplemented with 3% ALS mouse plasma and recombinant human apolipoprotein E (ApoE; Novus Biologicals, catalog #NBP1-99158) at a concentration of 50 μg/ml in duplicate. The next day, the LIVE/DEAD viability/cytotoxicity assay for cell viability was performed as described below.

##### Inhibition of PI3K/Akt/ApoA1 downstream signaling pathway

The mBECs were cultured in a 24-well plate, and the culture procedure was conducted as described above. The mBECs were then incubated in media supplemented with 3% ALS mouse plasma and ApoA1 (100 μg/ml) adding wortmannin (Sigma-Aldrich, catalog #W3144) at a concentration of 0.1 μm/ml (Deregibus et al., 2007) in duplicate. The next day, the LIVE/DEAD viability/cytotoxicity assay for cell viability was performed as described below.

#### Blocking ApoA1 integration

Blockage of ApoA1 integration into mBECs was performed via immunoprecipitation with human anti-ApoA1 goat polyclonal antibody (GeneTex, catalog #GTX27613). The mBECs were cultured in 24-well plate as described above. The mBECs were then incubated in media supplemented with 3% ALS mouse plasma and ApoA1 at a concentration of either 50 or 100 μg/ml. In another two experimental conditions, ApoA1 at either 50 or 100 μg/ml was immunoprecipitated by adding human anti-ApoA1 goat polyclonal antibody at 1:100 ratio (Hamzic et al., 2016) for 30 min before adding this cocktail into culture media. The next day, the LIVE/DEAD viability/cytotoxicity assay for cell viability was performed as described below. Experiments were conducted in duplicate.

To verify that adsorbance of ApoA1 with ApoA1 blocking antibody prevented incorporation of ApoA1 into mBECs, immunocytochemical staining for ApoA1, using primary anti-human recombinant rabbit monoclonal antibody (R&D Systems, catalog #MAB36641) in mBECs, was performed. After completion of blocking ApoA1, mBECs were fixed by 4% paraformaldehyde (PFA) in PBS solution. The cells were then preincubated in a blocking solution of 10% normal goat serum (NGS) and 3% Triton X-100 in PBS for 60 min at RT, followed by overnight incubation with rabbit monoclonal antibody (1 μg/ml) at 4°C. On the next day, cells were rinsed in PBS and incubated with goat anti-rabbit secondary antibody conjugated to rhodamine (1:700, Alexa, Invitrogen) for 2 h at RT. After several rinses in PBS, Vectashield containing DAPI (Vector Laboratories) was added in the culture wells. The cells were then examined under epifluorescence using an epifluorescent inverted Olympus IX71 microscope and images were taken for further analysis of fluorescent immunostaining. The study was performed in duplicate.

##### Coculture system

In a follow-up study, human bone marrow-derived endothelial progenitor cells (hBM-EPCs) were co-cultured with mBECs at a 1:3 ratio without cell contact using a similar procedure as described (Renaud and Martinoli, 2016; Nguyen et al., 2019). Briefly, cryopreserved hBM-EPCs (CELPROGEN) were thawed and centrifuged (100 × *g*/10 min) in PBS at RT. Cell viability was assessed before culture using the Vi-Cell cell viability analyzer (Beckman Coulter). Initially, hBM-EPCs (1 × 10^4^ cells/400 μl commercial basal media/insert) were cultured in an insert with 0.4-μm pore size (BD, catalog #353095) separately from mBECs (3 × 10^4^ cells/400 μl basal media/well) cultured with 3% ALS mouse plasma in a 24-well plate for 48 h. Then, an insert with hBM-EPCs was placed into the 24-well plate with mBECs and the coculture was incubated for an additional 48 h. After incubation, hBM-EPCs in an insert were fixed by 4% PFA in PBS solution and immunocytochemical staining for ApoA1 was performed using primary anti-human recombinant rabbit monoclonal antibody and goat anti-rabbit secondary antibody conjugated to rhodamine as described above. In a separate coculture set, blocking human anti-ApoA1 goat polyclonal antibody at 1:100 ratio was added to cultured mBECs supplemented with 3% ALS mouse plasma on placing an insert with hBM-EPCs in duplicate. The next day, the LIVE/DEAD viability/cytotoxicity assay for cell viability was performed as described below.

### Cell viability assay

Viability of cultured mBECs under exposure of different supplements was determined using the LIVE/DEAD viability/cytotoxicity kit (catalog #L3224, Fisher Scientific) as we previously described (Ehrhart et al., 2018; Garbuzova-Davis et al., 2020). Briefly, the culture media was removed and cells were washed with PBS twice in each well. The combined LIVE/DEAD assay reagents (375 μl) were added to each well and incubated at RT for 30 min. After incubation, randomly selected images (*n* = 5–7/well) were obtained at 20× magnification for cell quantification using an epifluorescent inverted Olympus IX71 microscope. Live cells were labeled with green fluorescence through the conversion of nonfluorescent cell-permanent calcein acetoxymethyl. Dead cells were identified using ethidium homo dimer-1, which enters cells through damaged membranes and produces a red fluorescence on binding to nucleic acids. Cell counts of live (green) and dead (red) cells were determined using NIH ImageJ software (version 1.46). The percentage of dead cells from total live/dead (green/red) cells was calculated from each image. Imaging analysis for each condition was performed by averaging results from 10–14 images.

### Obtaining plasma from mouse blood

Blood was obtained via submandibular bleeding of G93A SOD1 mutant male mice at 12–13 weeks of age at the early symptomatic stage (*n* = 7). These animals were used in our previous published studies (Garbuzova-Davis et al., 2017, 2019b). Briefly, mouse blood was collected in BD Microtainer blood collection tubes containing 1.0 mg K2EDTA anticoagulant (Becton, Dickinson and Company) and tubes remained at RT for 10 min. Samples were then centrifuged at 3000 rpm for 15 min at RT to separate blood cells and plasma. The plasma was then transferred to a new tube, aliquoted, and stored at −80°C.

### Statistical analysis

Data are presented as mean ± SEM. One-way ANOVA with *post hoc* Tukey’s HSD multiple comparison test was performed for statistical analysis using online statistical software (https://astatsa.com/; 2016 Navendu Vasavada). Significance was defined as *p* < 0.05.

## Results

### ApoA1 effects on ECs in a pathologic environment

**Figure 1.**
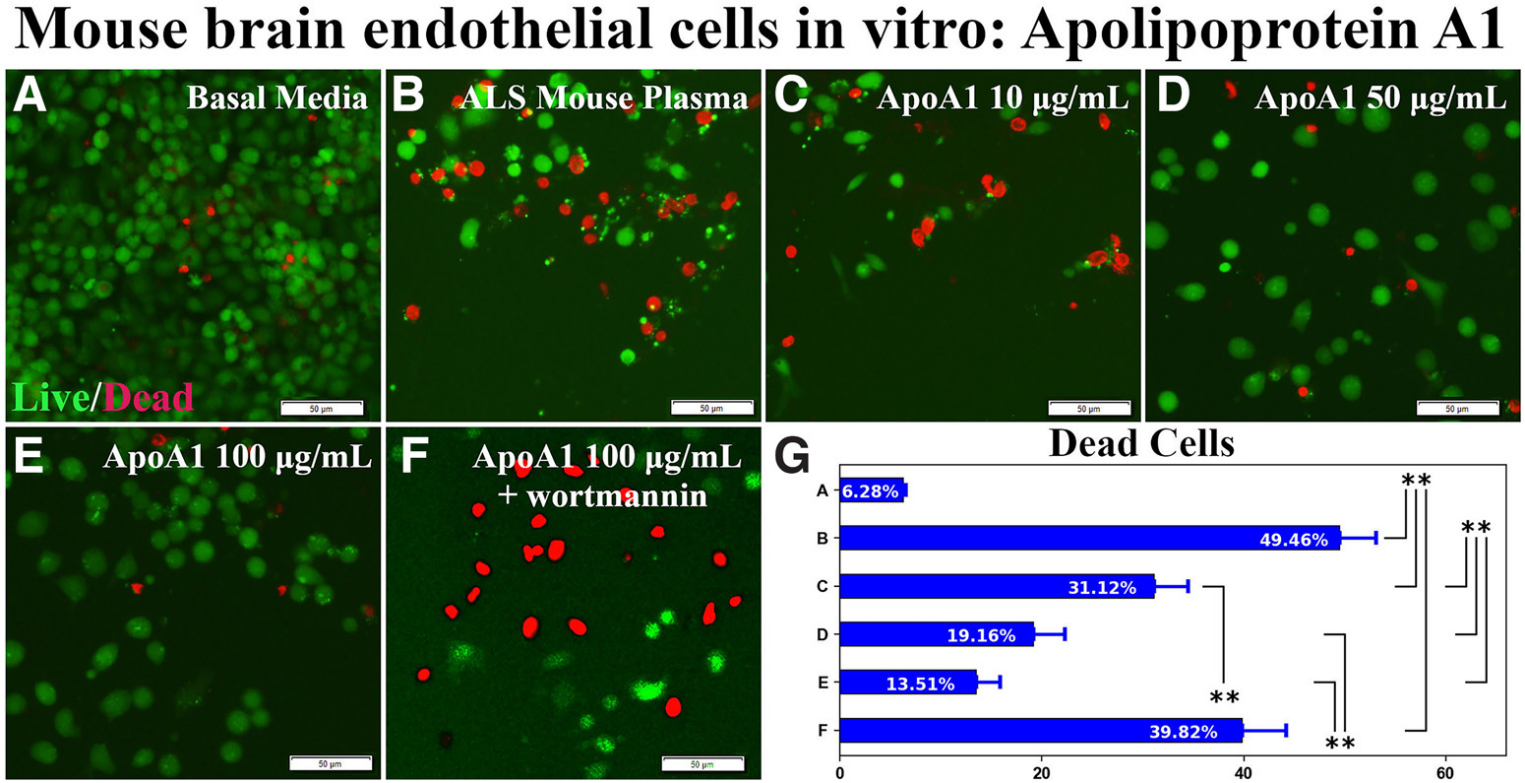
Effect of ApoA1 on mBEC viability after ALS mouse plasma exposure *in vitro*. ***A***, Numerous viable (green) mBECs were observed in basal media cultures. ***B***, Dead (red) cells significantly increased on exposure to 3% ALS mouse plasma. ***C*–*E***, Adding 10, 50, or 100 μg/ml of ApoA1 to culture media with ALS plasma, decreased dead cells in a dose-dependent manner. ***F***, Adding PI3K/Akt inhibitor wortmannin to ALS mouse plasma with 100 μg/ml ApoA1 significantly increased cell death. Scale bar: 50 μm (***A–F***). ***G***, Statistical analyses reflect imaging; **p < 0.01.

A dose-response *in vitro* study was performed to evaluate the effect of ApoA1 on mBECs exposed to 3% ALS mouse plasma. Data showed significantly (*p* < 0.01) increased mBEC death under media supplementation with ALS mouse plasma (49.46 ± 3.65%) compared with cells incubated in basal media (6.28 ± 0.28%; Fig. 1*A*,*B*,*G*). Adding ApoA1 to culture media at different concentrations demonstrated significantly (*p* < 0.01) decreased cell death with benefits correlating with increased dosage: 10 μg/ml (31.12 ± 3.37%), 50 μg/ml (19.16 ± 3.10%), and 100 μg/ml (13.51 ± 2.36%; Fig. 1*C–E*,*G*). Using the PI3K/Akt inhibitor, wortmannin, in combination with 100 μg/ml ApoA1 significantly (*p* < 0.01) elevated numbers of dead cells (39.82 ± 4.40%; Fig. 1*F*,*G*).

**Figure 2.**
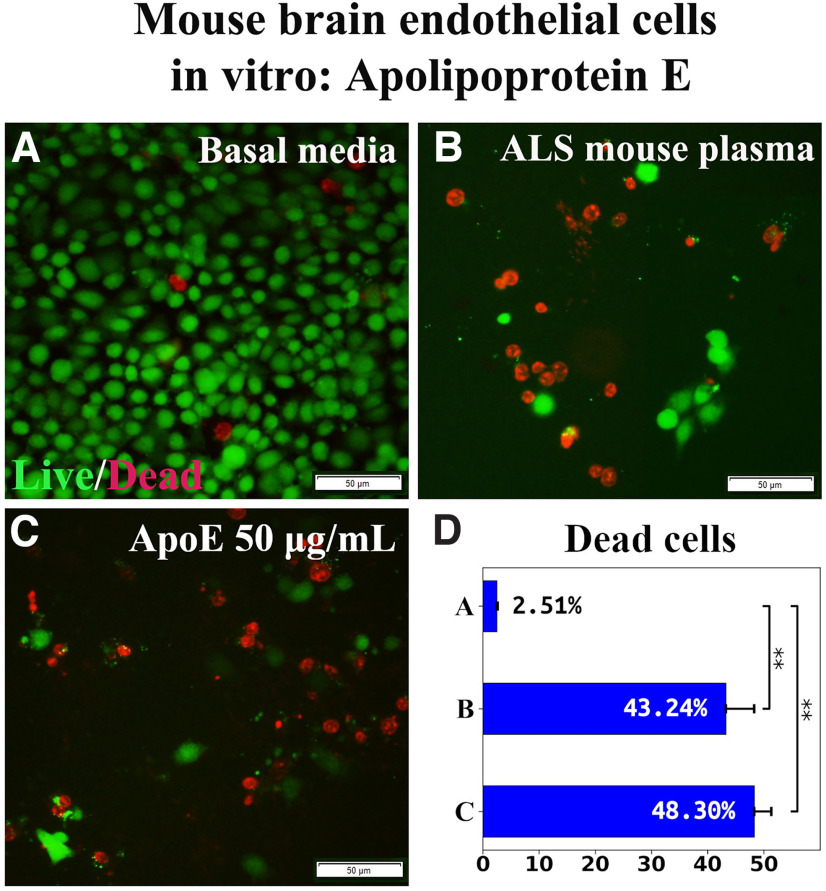
Effect of ApoE on mBEC viability after ALS mouse plasma exposure *in vitro*. ***A***, Numerous viable (green) mBECs were observed in cultures with basal media. ***B***, Dead (red) cells significantly increased after exposure to 3% ALS mouse plasma. ***C***, Adding 50 μg/ml of ApoE to culture media with ALS plasma did not reduce dead cells. Scale bar: 50 μm (***A***-***C***). ***D***, Statistical analyses reflect imaging; **p < 0.01.

To verify the specificity of ApoA1 effects, the consequences of ApoE exposure on mBECs were examined *in vitro*. ApoE at a concentration of 50 μg/ml was added to culture media supplemented with 3% ALS mouse plasma. Results showed that mBEC death under ApoE (48.30 ± 3.02%) nonsignificantly differed from the cell culture with ALS mouse plasma alone (43.24 ± 5.05%; Fig. 2*B–D*).

Together, these results showed that ApoA1 mediated EC survival under pathologic conditions and the effect was confirmed by inhibition of the PI3K/Akt downstream cytosolic signaling pathway. Also, ApoE was ineffective in ameliorating induced mBEC death, confirming specificity of ApoA1 in mediating EC survival.

### The effects of blocking ApoA1 integration into ECs

**Figure 3.**
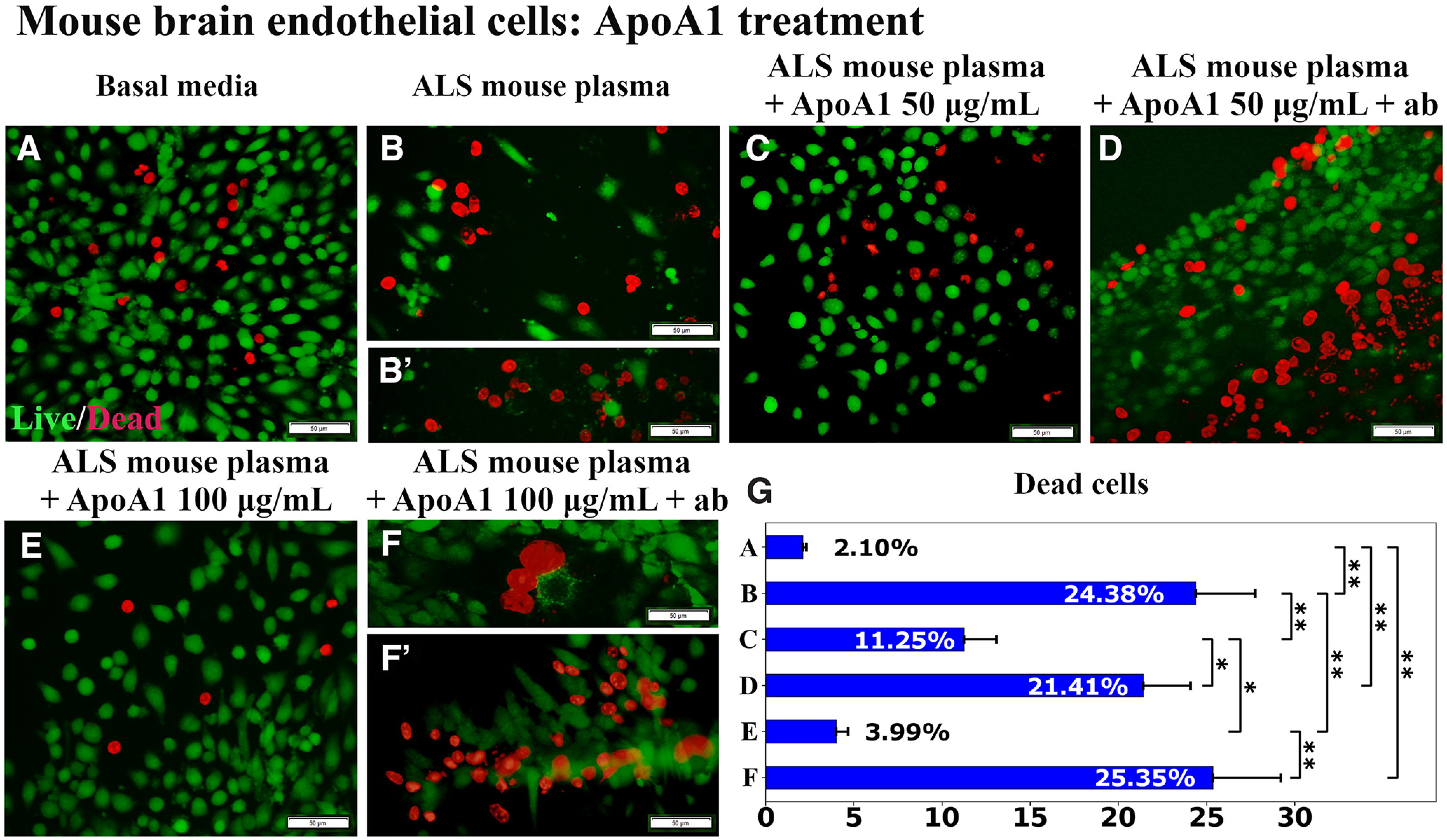
Immunoprecipitation of APOA1 with human anti-ApoA1 antibody. ***A***, Numerous viable (green) mBECs were observed in basal media cultures. ***B***, ***B’***, Dead (red) cells significantly increased on exposure to 3% ALS mouse plasma. Adding 50 μg/ml (***C***) or 100 μg/ml (***E***) of ApoA1 to culture media with ALS mouse plasma, significantly decreased dead cells. Blocking antibody added with 50 μg/ml (***D***) or 100 μg/ml (***F***, ***F’***) of ApoA1 to culture media with ALS plasma, significantly increased mBEC death. Scale bar: 50 μm (***A–F’***). ***G***, Statistical analyses reflect imaging; *p < 0.05, **p < 0.01.

In this experiment, ApoA1 was immunoprecipitated with human anti-ApoA1 antibody before adding to the culture of mBECs exposed to 3% ALS mouse plasma. Similar to previous study results, ApoA1 treatment at doses of both 50 μg/ml and 100 μg/ml significantly (*p* < 0.01) decreased mBEC death (11.25 ± 1.83% and 3.99 ± 0.70%, respectively; Fig. 3*C*,*E*,*G*) versus cell culture supplemented with ALS mouse plasma alone (24.38 ± 3.38%; Fig. 3*B*,*G*). Using the immunoadsorbed ApoA1, the protective effects of ApoA1 were lost, resulting in a substantial increase in cell death at the 50 μg/ml (21.41 ± 2.69%, *p* < 0.05) or 100 μg/ml (25.35 ± 3.86%, *p* < 0.01) dose (Fig. 3*D*,*F*, *F’*,*G*).

**Figure 4.**
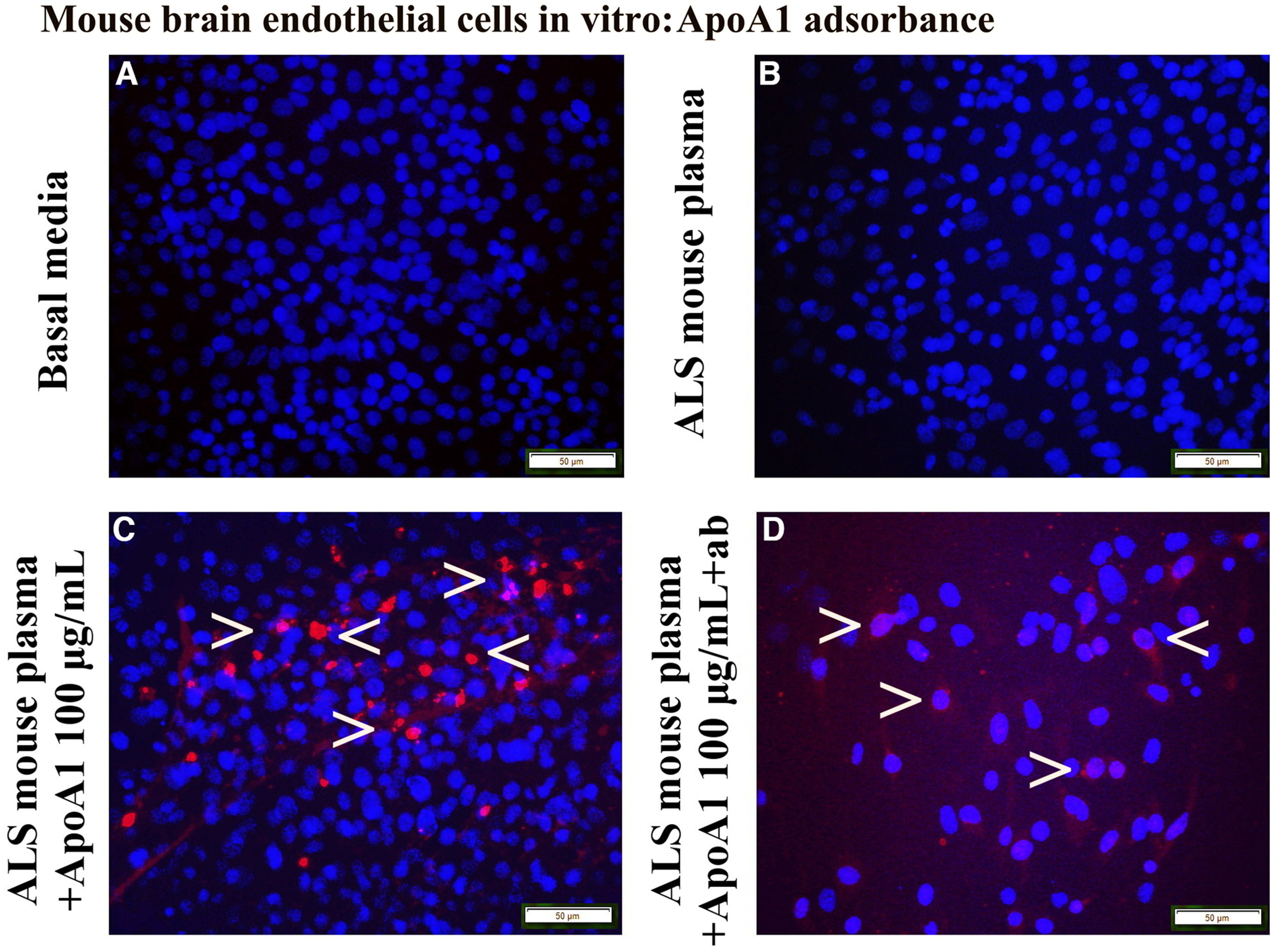
Immunocytochemical analysis of ApoA1 uptake into mBECs exposed to ALS mouse plasma and immunoadsorbed ApoA1. ApoA1 was not detected in cultured mBECs grown in basal media (***A***) or 3% ALS mouse plasma (***B***). ***C***, Numerous mBECs showed cytosolic ApoA1 immunostaining (red, arrowheads) after adding 100 μg/ml protein. After adding the ApoA1 blocking antibody cocktail with ALS plasma, few mBECs immunostained for ApoA1 (red, arrowheads) in culture with a 100 μg/ml ApoA1 dose (***D***). Scale bar: 50 μm (***A–D***).

To confirm ApoA1 integration into mBECs on exposure to 3% ALS mouse plasma, immunocytochemical staining for human ApoA1 using recombinant rabbit monoclonal antibody was performed *in vitro*. In this study, 100 μg/ml of ApoA1 was used because of demonstrating a more beneficial protective cell effect versus 50 μg/ml of protein in our above investigation. Results showed that mBECs cultured with basal media or ALS mouse plasma demonstrated negative ApoA1 immunostaining (Fig. 4*A*,*B*). After adding ApoA1 100 μg/ml to culture media with ALS mouse plasma, numerous mBECs immunoexpressed ApoA1 in cytosol (Fig. 4*C*). After immunoadsorbing 100 μg/ml of ApoA1 with blocking antibody and then adding to culture media with ALS plasma, fewer mBECs were positive for ApoA1 (Fig. 4*D*).

Thus, these results showed that ApoA1 incorporated into mBECs exposed to 3% ALS mouse plasma and this effect was confirmed by blocking ApoA1 cellular integration, thereby worsening EC survival.

### Coculture hBM-EPCs and mBECs

**Figure 5.**
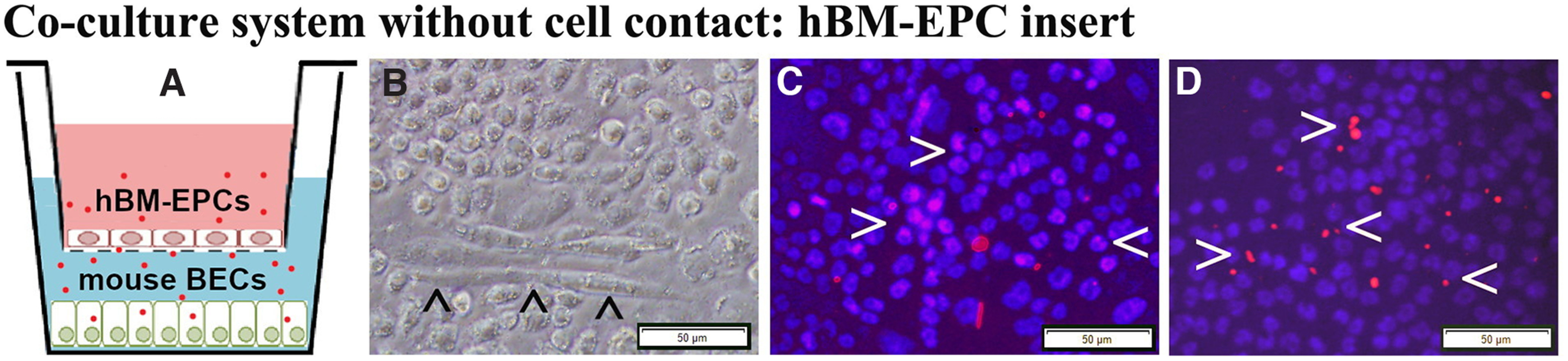
Co-cultured hBM-EPCs with mBECs and immunocytochemical validation of ApoA1 in hBM-EPCs. ***A***, Diagram of the coculture system without cell-to-cell contact. hBM-EPCs were cultured in an insert with 0.4-μm pore size with basal media (pink). mBECs were cultured in a 24-well plate with 3% ALS mouse plasma (blue). The red dots represent ApoA1 released into the culture media. ***B***, Phase-contrast image of live hBM-EPCs in insert; these cells displayed tubule-like morphology (arrowheads). Immunocytochemical staining of hBM-EPCs for ApoA1 showed immunopositive cells containing (***C***) and also releasing (***D***) ApoA1 protein (red, arrowheads). Scale bar: 50 μm (***B–D***).

**Figure 6.**
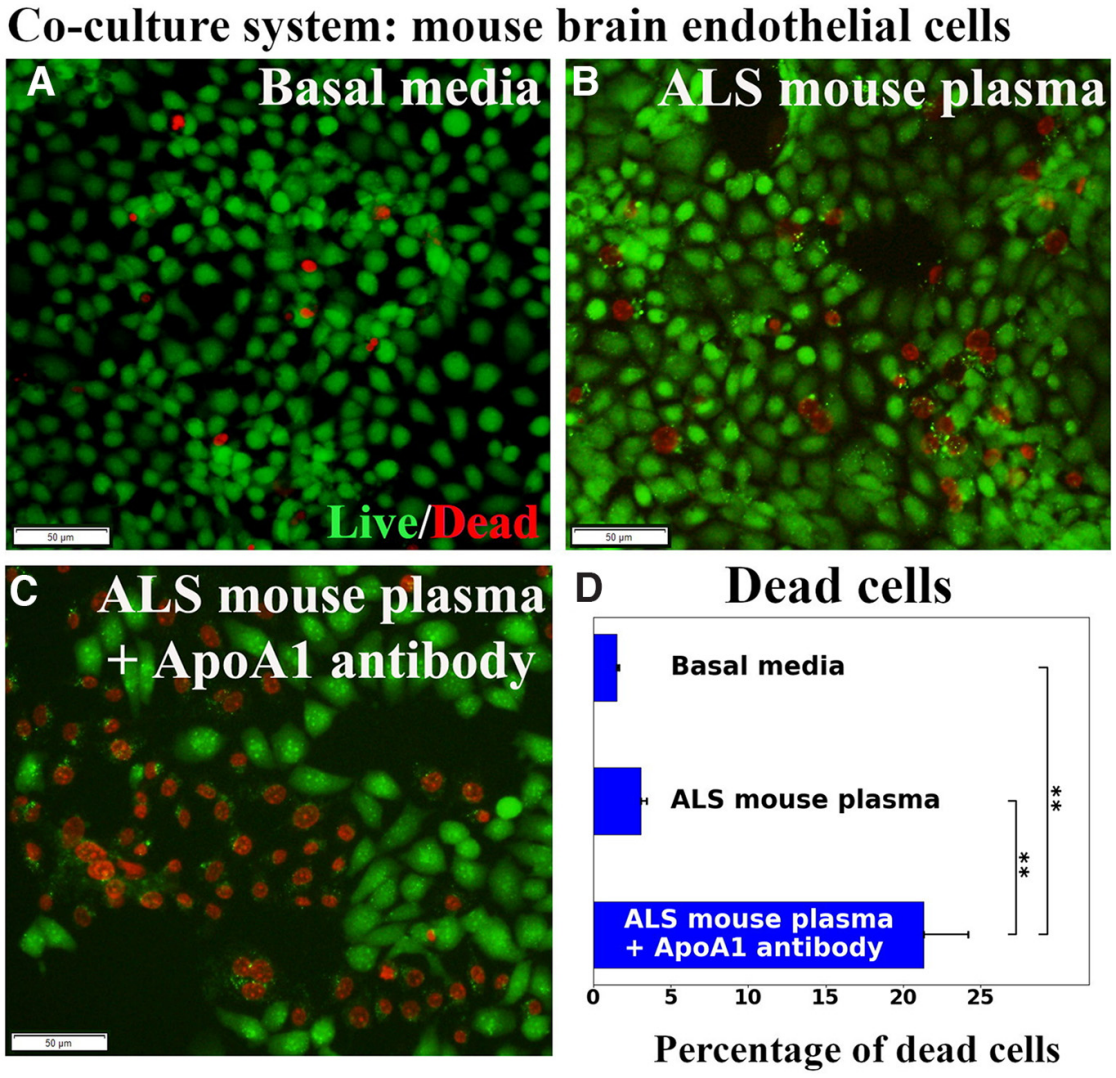
Evaluation of mBECs co-cultured with hBM-EPCs. No significant differences in mBEC death between cells cultured with basal media (***A***) or ALS mouse plasma (***B***) were observed. ***C***, Adding ApoA1 blocking antibody to culture media with ALS plasma, significantly increased mBEC death. Scale bar: 50 μm (***A–C***). ***D***, Statistical analyses reflect imaging; **p < 0.01.

The hBM-EPCs and mBECs were co-cultured without cell-to-cell contact (Fig. 5*A*) to validate that ApoA1 secreted by hBM-EPCs alleviates mBECs death from exposure to ALS mouse plasma. Results showed that hBM-EPCs in the insert developed cobblestone and tubule-like morphologies (Fig. 5*B*). Immunocytochemical staining for human ApoA1 showed that hBM-EPCs not only contained ApoA1 (Fig. 5*C*), but also released this protein (Fig. 5*D*). Next, mBEC survival after exposure to 3% ALS mouse plasma was evaluated for potential efficacy of ApoA1 secreted by hBM-EPCs. Data showed no significant differences in mBEC death between cells cultured with basal media (1.50 ± 0.16%) or ALS mouse plasma (3.07 ± 0.37%; Fig. 6*A*,*B*,*D*). When blocking ApoA1 antibody was added to culture media with ALS plasma, a significant (*p* < 0.01) increase of mBEC death (21.31 ± 2.89%) was determined (Fig. 6*C*,*D*).

Together, cell coculture results indicated that ApoA1 may be secreted by hBM-EPCs and this protein was essential for survival of ECs exposed to plasma from symptomatic G93A SOD1 mice.

## Discussion

In the present study, ApoA1 as a potential therapeutic for endothelium repair in ALS was characterized under a pathologic environmental condition *in vitro*. The major study findings showed that ApoA1: (1) significantly reduced mBEC death from pathologic environment; (2) mediated mBEC survival via the PI3K/Akt downstream cytosolic signaling pathway; (3) taken up by mBECs on exposure to a pathologic environment; and (4) was secreted by hBM-EPCs and incorporated into mBECs under exposure to a pathologic environment using a coculture system. These findings provide evidence that ApoA1 may be a potential novel therapeutic agent for protection of the endothelium in ALS by reducing cell death induced by the pathologic environment via protein incorporation into cells. Here, we show for the first time that targeting ApoA1 via an inhibitory approach supported the pivotal role of this protein as a key factor in development of an ALS treatment. Furthermore, results from this *in vitro* study show promise for ApoA1 as a therapeutic agent to restore the damaged endothelium in ALS.

Approximately 70% of HDL is composed of ApoA1 particles that are mainly referred to as anti-atherogenic lipoproteins, providing anti-inflammatory, antioxidative, and antithrombotic actions (Tabet and Rye, 2009; Smith, 2010; Walldius, 2012; Cochran et al., 2021). Based on reported positive ApoA1 effects to reduce EC-induced cytotoxicity (Svensson et al., 2017) or carotid artery inflammation (Puranik et al., 2008), the current study was designed to determine whether ApoA1 alleviates injury to ECs under a pathologic *in vitro* environmental condition reminiscent of ALS. We demonstrated damaged ECs in the CNS in both ALS patients (Garbuzova-Davis et al., 2012) and an animal model of disease (Garbuzova-Davis et al., 2007a,b). Other researchers (Henkel et al., 2009; Miyazaki et al., 2011; Winkler et al., 2013) have also demonstrated impairment of the blood-CNS-barrier in ALS patients. Barrier alterations were identified by examination of postmortem tissue from the brain and spinal cord of ALS patients. Although this barrier damage may occur at early disease stage, the timing or cause of EC degeneration in ALS is still undetermined. One possibility is that endogenous ECs, lining the CNS capillary lumen and facing the blood circulation, can be affected by the pathologic humoral environment in ALS. A meta-analysis of various cytokines in G93A SOD1 mutant mice from 66 peer-reviewed studies demonstrated increased levels of proinflammatory Type I cytokines versus anti-inflammatory Type II cytokines across all disease stages (Jeyachandran et al., 2015). Moreover, analysis of 97 cytokines in plasma from G93A SOD1 mutant mice at different disease stages showed increased levels of several cytokines that were associated with a shorter mouse lifespan (Moreno-Martínez et al., 2019). In addition, proinflammatory cytokines in plasma from ALS mice may trigger EC apoptosis. However, studies to determine ALS plasma-induced apoptosis by establishing specific molecular profile of apoptotic signals have yet to be addressed.

Mimicking a pathologic *in vivo* environment, mBECs were exposed *in vitro* to plasma obtained from early symptomatic G93A SOD1 mutant mice. The choice of 3% ALS mouse plasma was based on results of our previous study (Garbuzova-Davis et al., 2020), showing moderate cell damage from this dosage versus 1% or 5% ALS mouse plasma. Exposure to 3% ALS mouse plasma led to significantly increased mBEC death, similar to previous results (Garbuzova-Davis et al., 2020), suggesting the influence of the pathologic environment on cell survival. The above-mentioned study also showed no significant differences in mBEC viability between cells cultured in basal media and those supplemented with control mouse plasma. In the current study, mBECs were incubated in the same media with different supplements and cell viability (LIVE/DEAD assay) was compared with cells cultured under normal condition (basal media). Since mBECs were cultured under the same condition in terms of the basal media, using this media may serve as an appropriate control to determine cellular status under supplementations. Additionally, our culture design allowed suitable reproducibility for comparative cellular analyses, specifically under ApoA1 treatment.

A dose-response study established that ApoA1 substantially decreased cell death with most pronounced reduction at a concentration of 100 μg/ml. When wortmannin, a PI3K/Akt inhibitor, was added to culture media in combination with ApoA1, significantly elevated numbers of dead mBECs were observed. Thus, these results showed an advantageous ApoA1 effect on mediating EC survival under pathologic condition and this effect was confirmed by inhibition of the PI3K/Akt downstream cytosolic signaling pathway. Possible beneficial effects related to anti-inflammatory ApoA1 actions were discussed in recent reviews (Jackson et al., 2021; Robert et al., 2021). However, to confirm this suggestion, proinflammatory and anti-inflammatory cytokine content in induced mBEC death before and after ApoA1 treatment needs to be elucidated. We are planning such a study for the near future. Additionally, activation of the Akt-dependent pathway, critical for angiogenesis and development of human microvascular ECs into capillary-like structures, was prevented by PI3K inhibitor wortmannin *in vitro* at the same concentration of 0.1 μm/ml (Deregibus et al., 2007), supporting our study findings. On the other hand, ApoA1 induced *in vitro* proliferation and angiogenesis of human EPC obtained from peripheral blood of healthy subjects through activation of the surface ecto-F1-ATPase receptor (González-Pecchi et al., 2015). Yet, the current study did not focus on detecting the effect of ApoA1 on mBEC proliferation or angiogenic capacity and we intend to address these points in the near future.

We performed another experiment to examine ApoA1’s input on eliminating mBEC death on a pathologic condition by blocking ApoA1 integration into cultured cells exposed to ALS mouse plasma via immunoprecipitation with human anti-ApoA1 polyclonal antibody. This antibody was successfully used to verify ApoA1’s role as a biomarker of aplastic anemia by selectively immunodepleting this protein from patient serum (Hamzic et al., 2016). Our study results provided convincing evidence of a significant increase in mBEC death by blocking ApoA1 with an antibody. Moreover, ApoA1 integration into mBECs was confirmed by immunocytochemistry showing that a number of cells immunoexpressed ApoA1 in cytosol and addition of the blocking antibody prevented ApoA1 uptake into the mBECs. Thus, the study findings showed that ApoA1 incorporated into mBECs on exposure to ALS mouse plasma and this effect was confirmed by blocking ApoA1 cellular integration. However, mBECs were cultured in basal media containing 10% FBS, which might influence lipid content. FBS contains the various proteins, lipids, hormones, enzymes, etc., necessary for normal cell growth. Since mBECs were cultured at the same condition in terms of FBS, the effects of media supplements (ALS mouse plasma, ApoA1, or specific antibodies) were clearly demonstrated in Figure 3. However, cells cultured in serum free media could be appropriate controls to clarify our study results. Also, ApoA1 was obtained from human plasma, which does not interfere with FBS. Testing ApoA1 amounts in culture media by western blotting might confirm blockage of this protein’s integration into cells via antibody immunoprecipitation. These studies are planned for the near future.

In a separate study, ApoE’s effect on mBEC survival under a pathologic condition was examined *in vitro* and demonstrated that this apolipoprotein was not beneficial in ameliorating induced cell death, confirming the specificity of ApoA1 in mediating EC survival. However, the role of ApoE in lipoprotein metabolism of various neurodegenerative diseases needs further discussion. ApoE is a well-known risk factor for Alzheimer’s disease (Armstrong, 2019; Muñoz et al., 2019; Chen et al., 2021) and Parkinson’s disease (Huang et al., 2004; Y.J. Li et al., 2004a; Davis et al., 2020). In ALS, studies regarding a link between the ApoE genotype and the risk of developing disease are contradictory. Early studies showed that ApoE-4 allele is not associated with age at onset and site of onset in ALS (Mui et al., 1995; Moulard et al., 1996; Bachus et al., 1997; Siddique et al., 1998). But it has been shown that the ApoE-2 allele is protective against early ALS onset, suggesting that this apolipoprotein “may express its strongest effect through age at onset rather than on risk” (Y.J. Li et al., 2004b). Another study reported an opposing role of ApoE-2 allele as a risk factor for cognitive impairment in ALS, signifying a link between cholesterol hypometabolism and neurodegeneration (Canosa et al., 2019). Despite inconsistent reports of ApoE’s role in ALS, it is possible that the ApoE-4 allele is a genetic risk factor and the ApoE-2 allele is a genetic protective factor for disease development, similar to the situation with Alzheimer’s disease (for review, see Serrano-Pozo et al., 2021). However, ApoE’s genome-wide association with ALS needs to be clarified. In our current study, there are at least two possible reasons that ApoE was ineffective in ameliorating mBEC survival under a pathologic condition. First, we used recombinant human ApoE without allelic specificity. Second, ApoE may not have integrated into ECs *in vitro*. These suppositions require confirmation.

Finally, we are continuing to investigate the mechanisms by which hBM-EPCs, intravenously transplanted into symptomatic G93A SOD1 mutant mice, benefit restoration of the blood-CNS-barrier, leading to improved disease outcomes and increased motor neuron survival (Garbuzova-Davis et al., 2019b). To mimic an *in vivo* condition, hBM-EPCs and mBECs were co-cultured without direct cell-to-cell contact. Our results showed that ApoA1 secreted by hBM-EPCs alleviated mBEC death from exposure to ALS mouse plasma via this lipoprotein’s incorporation into cells. Thus, these study results provide important evidence for therapeutic efficacy of hBM-EPC transplants through ApoA1 secretion. ApoA1’s beneficial role in EC survival, identified in the current study, along with secretion of angiogenic factors such as VEGF-A and angiogenin-1, by hBM-EPCs (Garbuzova-Davis et al., 2019a), are potential mechanisms for endothelium repair via cell therapy in ALS. Of note, the link between angiogenesis and cell survival in pathogenesis of ALS was discussed and emphasized that targeting specific angiogenic substances can extend the survival rate of ALS patients (for review, see Thakur et al., 2020). Since angiogenin and/or VEGF play important roles in promoting angiogenesis or vasculogenesis (Hoeben et al., 2004; Tammela et al., 2005; Gao and Xu, 2008; Crivellato, 2011; Vandekeere et al., 2015), these proteins may be considered as controls for ApoA1 in treatment of G93A SOD1 mice in our continuing *in vivo* research.

In summary, we conducted an *in vitro* study to determine ApoA1’s effects on mBECs under a pathologic environmental condition reminiscent of ALS, based on the well-known endothelial degeneration found in an animal disease model and in ALS patients. ApoA1, as the most abundant HDL protein, effectively reduced cell death via integration into cells. This effect was confirmed by inhibition of the PI3K/Akt downstream cytosolic signaling pathway and ApoA1 blocking antibody. Importantly, our coculture study demonstrated that ApoA1 was secreted by hBM-EPCs and incorporated into mBECs on exposure to ALS mouse plasma, confirming the therapeutic effect of hBM-EPC transplantation for EC repair observed in an ALS mouse model. However, expression and/or secretion of ApoA1 by hBM-EPCs needs confirmation via western blotting. Since altered lipoprotein metabolism was noted in ALS, our study findings provide evidence for the first time that ApoA1 may be a potential novel therapeutic agent for protection of the endothelium in this devastating disease. Although this *in vitro* study shows promise for ApoA1 as a therapeutic, further research is needed to determine ApoA1’s effects *in vivo* for restoration of the damaged endothelium in ALS. Specifically, defining the best ApoA1 dose on survival of G93A SOD1 mice treated presymptomatically and/or postsymptomatically will determine potential benefits of this protein during disease outcomes. Additionally, competence of neuro-vascular unit in G93A mice on ApoA1 treatment requires evaluation. This study might be imperative not only to confirm integrity of CNS barrier, but also to determine whether ApoA1 promotes neuronal cell survival. Also, the positive effect of ApoA1 on reduction of mBEC death that occurred via the PI3K/Akt downstream signaling pathway requires more evidence. These points will be addressed in our planned follow-up studies. Furthermore, more efforts should be focused on understanding the mechanisms of the lipid alterations affecting ECs and the specific role of ApoA1 in ECs restoration for development of targeted therapies for ALS.

## Synthesis

Reviewing Editor: Christopher Colwell, UCLA School of Medicine Decisions are customarily a result of the Reviewing Editor and the peer reviewers coming together and discussing their recommendations until a consensus is reached. When revisions are invited, a fact-based synthesis statement explaining their decision and outlining what is needed to prepare a revision will be listed below. The following reviewer(s) agreed to reveal their identity: Krishna Aluri, Deanna Smith.

ALS is a fatal neurodegenerative disease with no cure. We are still understanding the mechanisms of pathogenesis. Both reviewers agreed that this study makes important contributions.

The authors did a really good job understanding the role of ApoA1 protein involvement in the survival of endothelial cells in vitro. The authors showed exposure mBECs to ALS G93A mouse plasma caused cell death and showed the cells can be rescued by treatment with ApoA1 in a dose-dependent manner. They further demonstrated the specificity by blocking ApoA1 integration using antibodies.

Additional experiments:

While this data shows a promising role of ApoA1 in endothelial cell survival in vitro, this study overlooks the role of ApoA1 in overall disease pathogenesis.

To further substantiate the results and better understand the role of ApoA1 in treating ALS, we recommend the authors to perform a survival study in G93A mice dosed with ApoA1 in both pre and post-symptomatic mice. It has been shown that Angiogenin another protein causing ALS plays a crucial role in angiogenesis has a paradoxical effect when use pre and post symptomatically and delays disease onset when G93A mice are treated presympotmatically) a similar study using G93A mice would be quite beneficial. We feel that this would greatly strengthen the study.

The authors did not comment on the role of ApoA1 on the survival of other neuronal cells involved in ALS pathogenesis such as neurons and astrocytes. It will add to current data if they can further study the effect on the mentioned cell types.

The appropriate control for G93A-SOD1 mouse plasma is be plasma from a normal mouse, not basal media.

Are there candidate apoptotic signals in the plasma from the ALS mouse model that might be triggering apoptosis? If so, could this be tested using the mBEC LIVE/DEAD assay?

What is the lipid/Apo profile in FBS? The experiments might have been best carried out in serum free medium rather than simply adding serum from G93A-SOD1 mouse to mBEC cells cultured in the presence of 10% FBS, especially when ApoA1 is theoretically removed by immunoprecipitation (Figure 3). At a minimum the rationale for this should be discussed, and the amount of ApoA1 in cell culture media tested by western blotting.

Other questions to be addressed by authors:

The authors used 3% mouse plasma for their in vitro experiments how did they decide on this dose? Did they use any other concentrations?

The significance of the findings with respect to ALS need to be clarified. What are the vascular changes that occur in patients? There is a reference to the authors paper (Garbuzova-Davis 2012) that indicated an observed endothelial degeneration in ALS brains - this needs to be expounded upon and discussed more thoroughly in the context of disease progression - especially in the discussion. The study they reference to support more dyslipidemia in ALS patients (Cheslowska, 2021) did not find correlates with disease progression rate, only disease duration this needs further discussion as well.

Also, if ALS patients have elevated ApoA1 levels, do the authors consider this a protective adaptation to serum factors that may damage endothelial cells in ALS plasma?

Figure 3:It is not clear why there is a B and B’ panel, and an F and F’ panel. Do these represent two views of the same treatment, or is there a difference?

Figures 5 and 6: The rationale for coculturing with human bone-marrow derived endothelial progenitor cells was not clear. Are these cells known producers of ApoA1 in serum?

Figure 5D: it is not clear the red puncta represent released ApoA1. Western blots from co-culture media should be compared to media from mBECs only.

Figure 6C: Was the antibody used to clear ApoA1 from the coculture media as indicated for Figure 3, or was it simply added to the media?
