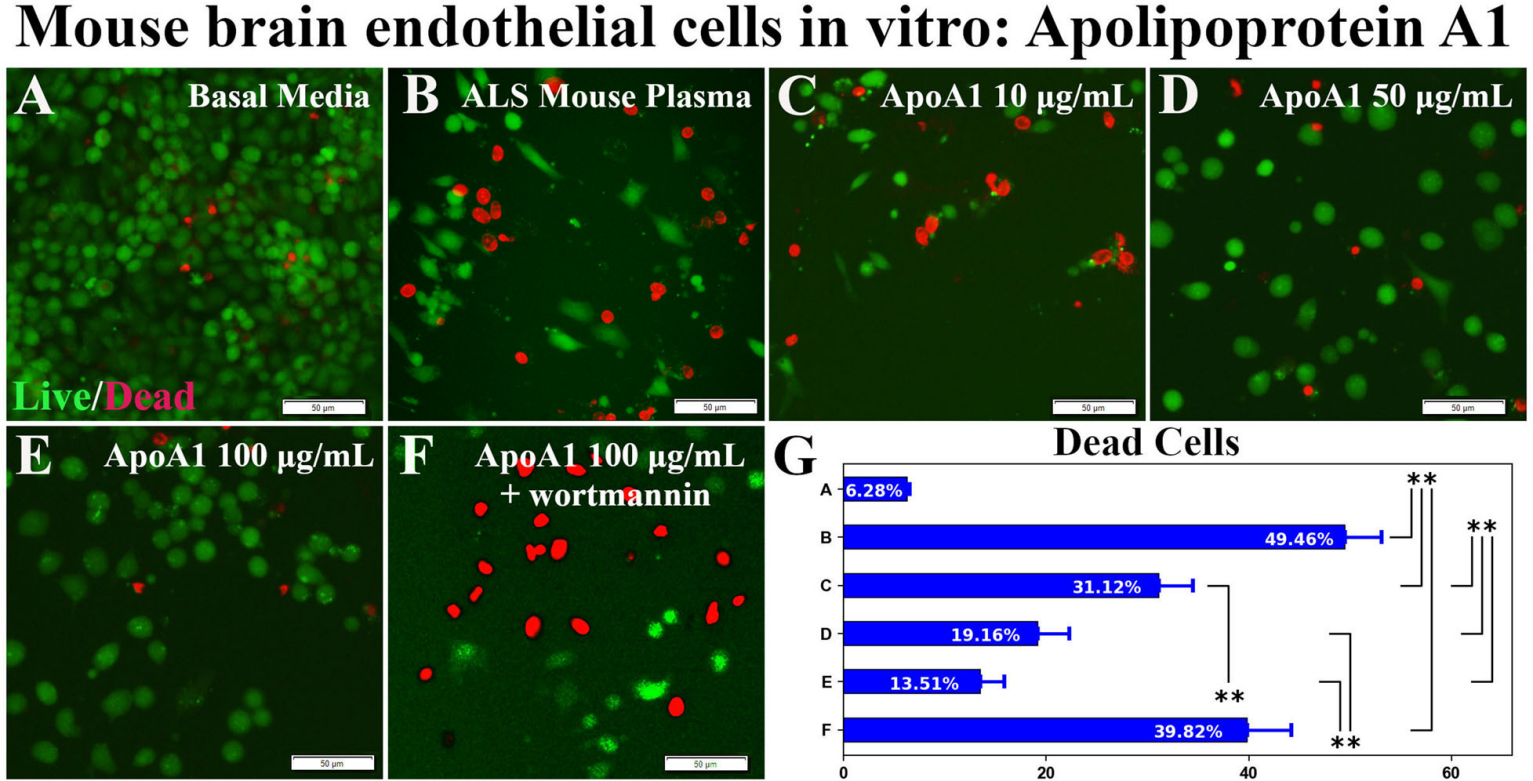# Erratum: Garbuzova-Davis et al., “Apolipoprotein A1 Enhances Endothelial Cell Survival in an In Vitro Model of ALS”

**DOI:** 10.1523/ENEURO.0280-24.2024

**Published:** 2024-07-10

In the article “Apolipoprotein A1 Enhances Endothelial Cell Survival in an In Vitro Model of ALS,” by Svitlana Garbuzova-Davis, Alison E. Willing, and Cesario V. Borlongan, published online on July 15, 2022, Figure 1 was published with an error. During final figure assembly, Figure 1*B* was taken from another study the authors were conducting involving an ALS plasma-treated model. A corrected version of Figure 1 appears below. All authors have reviewed the images and raw data associated with the study and confirm that this error does not affect the conclusions of the paper.

**Figure 1.**